# Counseling With Guided Use of a Mobile Well-Being App for Students Experiencing Anxiety or Depression: Clinical Outcomes of a Feasibility Trial Embedded in a Student Counseling Service

**DOI:** 10.2196/14318

**Published:** 2019-08-15

**Authors:** Emma Broglia, Abigail Millings, Michael Barkham

**Affiliations:** 1 Research Department British Association for Counselling and Psychotherapy Leicestershire United Kingdom; 2 Department of Psychology University of Sheffield Sheffield United Kingdom

**Keywords:** counseling, students, mental health, mobile app, feasibility studies, outcome measures, depressive symptoms, generalized anxiety, universities

## Abstract

**Background:**

Anxiety and depression continue to be prominent experiences of students approaching their university counseling service. These services face unique challenges to ensure that they continue to offer quality support with fewer resources to a growing student population. The convenience and availability of mobile phone apps offer innovative solutions to address therapeutic challenges and expand the reach of traditional support.

**Objective:**

The primary aim of this study was to establish the feasibility of a trial in which guided use of a mobile phone well-being app was introduced into a student counseling service and offered as an adjunct to face-to-face counseling.

**Methods:**

The feasibility trial used a two-arm, parallel nonrandomized design comparing counseling alone (treatment as usual, or TAU) versus counseling supplemented with guided use of a mobile phone well-being app (intervention) for 38 university students experiencing moderate anxiety or depression. Students in both conditions received up to 6 sessions of face-to-face counseling within a 3-month period. Students who approached the counseling service and were accepted for counseling were invited to join the trial. Feasibility factors evaluated include recruitment duration, treatment preference, randomization acceptability, and intervention fidelity. Clinical outcomes and clinical change were assessed with routine clinical outcome measures administered every counseling session and follow-up phases at 3 and 6 months after recruitment.

**Results:**

Both groups demonstrated reduced clinical severity by the end of counseling. This was particularly noticeable for depression, social anxiety, and hostility, whereby clients moved from elevated clinical to low clinical or from low clinical to nonclinical by the end of the intervention. By the 6-month follow-up, TAU clients’ (n=18) anxiety had increased whereas intervention clients’ (n=20) anxiety continued to decrease, and this group difference was significant (Generalized Anxiety Disorder–7: *t*_22_=3.46, *P*=.002). This group difference was not replicated for levels of depression: students in both groups continued to decrease their levels of depression by a similar amount at the 6-month follow-up (Physical Health Questionnaire–9: *t*_22_=1.30, *P*=.21).

**Conclusion:**

Supplementing face-to-face counseling with guided use of a well-being app is a feasible and acceptable treatment option for university students experiencing moderate anxiety or depression. The feasibility trial was successfully embedded into a university counseling service without denying access to treatment and with minimal disruption to the service. This study provides preliminary evidence for using a well-being app to maintain clinical improvements for anxiety following the completion of counseling. The design of the feasibility trial provides the groundwork for the development of future pilot trials and definitive trials embedded in a student counseling service.

**Trial registration:**

ISRCTN registry ISRCTN55102899; http://www.isrctn.com/ISRCTN55102899

## Introduction

Identifying the prevalence of mental ill-health in university students has been a longstanding priority of educational institutions, and the growing concern over student mental health is widespread [1-3]. Research reports collating global accounts of student mental ill-health have found that up to 74% of students experience moderate emotional distress by the second semester, and this, in turn, has been linked to negative outcomes including low academic performance, isolation, financial problems, and time away from education leading to depression [4-7]. In recent years, the World Mental Health Survey of 13,984 full-time students from 19 colleges across 8 countries found that 35% met the threshold for at least one of the following common lifetime mental disorders (according to the *Diagnostic and Statistical Manual of Mental Disorders, 4th Edition*, or DSM-IV): major depression, mania, generalized anxiety disorder, alcohol abuse, substance misuse, or panic disorder [8]. The survey also found that 75% of the students would not seek help for an emotional or mental health concern, with the most prominent reasons being that students would rather tackle the problem alone or seek help from friends or they feel too embarrassed to seek professional help [9]. An earlier study spanning 21 countries found that only 16% of 6452 students with diagnosable mental health disorders received the minimum adequate treatment, and this percentage decreased proportionally according to the income level of the country [10].

Research into the prevalence of mental ill-health in students has gained national attention, but there is limited recent data. In 2013, the National Union of Students (NUS) surveyed 1336 UK university students and found that the most common factors contributing to mental distress were academic deadlines, academic performance, and work-life balance [11]. Regarding help-seeking, 26% of students did not discuss their mental health concerns and of those who did, the most popular contacts were friends, family, their doctor, or an academic. Only 10% approached the university counseling service compared with 17% to 21% who approached and used advice from their union. Between 2014 and 2015, the Higher Education Funding Council for England commissioned a report to identify the mental health needs of students and found increased declarations for student disability on the basis of mental ill-health [12]. The report identified increased academic staff time being used to address student mental health as well as therapeutic staff managing larger caseloads and seeing more students with complex or comorbid concerns for anxiety, depression, and self-harm.

The challenges to addressing student mental health in UK higher education institutions have been characterized by comparing 113 in-house student counseling services [13]. Increased demand for counseling, drop out between counseling sessions, and growing waiting lists are some of the critical issues that student counseling services face. While not unique to the education sector, these challenges, along with sector-specific challenges of changes to the funding climate and disruptions from academic timetables [14,15], have hindered the development of robust research, rendering the evidence base for student counseling limited and pathways to improve service provision unclear. That said, many higher education institutions are either offering or are interested in offering digital solutions as a way of attempting to do more for less [13] and make their limited resources reach further. The use of technology in student counseling services has steadily grown over time, and the types of technology being used have changed in line with the availability of new technology and evidence. Early applications of technology in therapy altered the format in which counseling was traditionally delivered, offering counseling via telephone, email, and videoconference as a way of extending face-to-face support to clients unable to attend the counseling service [16].

As the application and convenience of technology have advanced, the use of digital technology offers innovative ways to address therapeutic challenges and expand the reach of traditional support. The most commonly used digital mental health tools today are Web- and phone-based apps, both of which are widely used and recommended in national health services, including the National Health Service (NHS) in England [17]. Evidence for these therapeutic technologies is steadily growing, and Web-based interventions for anxiety, depression, and stress can be effective in students [18]. Web-based interventions appear particularly effective for anxiety and depression, leading to significant reductions in symptom severity with small to medium effect sizes that are maintained long term [19]. These findings are consistent with a meta-analysis of 16 clinical trials comparing internet-based cognitive behavioral therapy (iCBT) versus waitlist control groups, demonstrating less worsening of symptoms for intervention groups [20]. Such evidence has been heavily weighted toward studies of the general population rather than students. However, research involving students has emerged in recent years and highlights a shift toward evaluating the role of digital technology in improving student mental health outcomes. One randomized controlled trial (RCT) established the efficacy of an unguided internet intervention for 200 university students diagnosed with social anxiety disorder [21]. Following a 10-week self-guided text-based intervention, moderate to large effects were shown to reduce social phobia (*d*=0.76, 95% CI 0.47-1.04), depression (*d*=0.50, 95% CI 0.22-0.78), and fear of positive evaluation (*d*=0.27, 95% CI 0.01-0.55) and improve quality of life (*d*=0.41, 95% CI 0.13-0.69).

A meta-analysis of mental health and well-being internet interventions for university students evaluated 48 RCTs and identified small effects on stress (*g*=0.20, 95% CI 0.02-0.38) and depression (*g*=0.18, 95% CI 0.08-0.27), with moderate effects on disordered eating (*g*=0.52, 95% CI 0.22-0.83) [22]. These effects were particularly high for interventions that were 4 to 8 weeks long and based on CBT and when compared to control groups receiving no intervention. These findings show potential for internet interventions in the context of student mental health as in-house support services typically offer short-term support in the first instance. While promising, this meta-analysis also demonstrates the need for research to explore broader applications of internet interventions outside the remit of CBT to be more inclusive of the range of mental health interventions available at universities (eg, counseling) and expand the evidence base to include research designs that go beyond comparisons with no intervention. Moreover, recent years have seen the rapid development of smartphone-based apps, which are arguably more suited to the student lifestyle. Smartphone apps can be effective for treating anxiety [23] and depression [24], but little is known about the perception, application, or potential benefits of augmenting student counseling services with apps.

There is a significant need to explore innovative solutions to ensure that counseling services continue to offer quality support to a growing student population while being under continual threat of reduced resources. Augmenting counseling with apps (ie, blended intervention) may provide one such solution, and a feasibility trial is required to examine the feasibility and acceptability of doing so. Recent studies exploring the determinants of blended approaches have shown that therapists perceive the possibilities of adding blended work to their routine practice, blended approaches may help to reduce treatment gaps, there are training opportunities for offering mobile apps in clinical settings, clients can experience apps to be supportive of face-to-face interventions, and blended approaches offer potential to reach more patients than face-to-face alone [25-28]. There is a need to explore the feasibility of offering blended interventions to university students and to do so in a natural setting without disrupting the service or denying access to support. Accordingly, the primary aim of this study was to report on the outcomes of a feasibility trial in which guided use of a mobile phone well-being app was introduced into a student counseling service and offered as an adjunct to face-to-face counseling [29]. This primary aim was assessed by a range of feasibility factors including recruitment, treatment preference, and randomization acceptability. The secondary aim was to examine clinical outcomes and clinical change.

## Methods

### Trial Design and Setting

**Figure 1:**
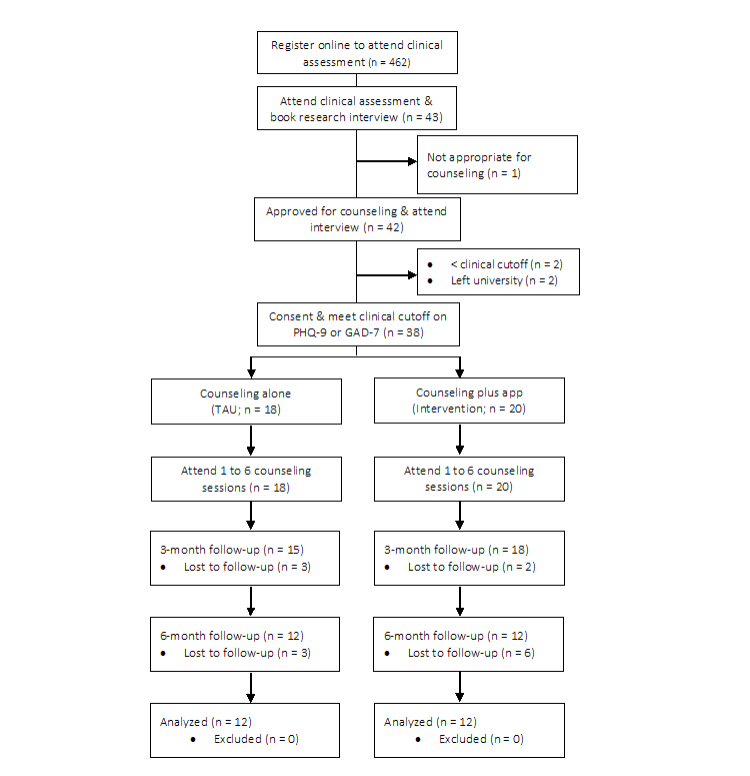
Participant flow diagram summarizing the number of clients that were recruited for the feasibility trial, were eligible, and participated at 3-month and 6-month follow-up phases. PHQ-9: Patient Health Questionnaire, 9-item; GAD-7: Generalized Anxiety Disorder, 7-item; TAU: treatment as usual.

This feasibility trial used a two-arm, parallel, nonrandomized design comparing counseling alone (treatment as usual, or TAU) versus counseling supplemented with guided use of a well-being app and discussion of app activities (intervention) for university students experiencing anxiety or depression (Figure 1). The feasibility trial was registered on the ISRCTN registry [ISRCTN55102899] in 2016 under the acronym CASELOAD (Counseling Plus Apps for Students Experiencing Levels of Anxiety or Depression) and has been published elsewhere [29]. The study received ethical approval from the University of Sheffield Department of Psychology Research Ethics Committee on January 5 2016 (ref: 006171) and underwent independent scientific review. The trial was embedded in the university counseling service with high stakeholder engagement from the head of service and therapeutic team.

### Participants and Procedure

Participants were 38 help-seeking university students (aged 18 years and over) who had been accepted for counseling and met moderate clinical criteria on one of two standardized outcome measures for anxiety or depression. Inclusion criteria also included (1) newly registered for counseling, (2) undergraduate or postgraduate, and (3) having access to a personal smartphone with ability to install a publicly available app (iOS or Android). Participants were excluded if they met any of the following criteria: (1) present with a high risk to self or others, (2) already receiving therapeutic support, or (3) complex mental health problems beyond anxiety and/or depression. In line with routine practice, students who approached the counseling service were assessed by a therapist to determine their appropriateness for counseling. Students approved for counseling were invited to attend a 20-minute research interview to determine their eligibility. Students who attended the research interview were asked to provide written informed consent and were assessed for eligibility through completion of the standardized outcome measures. Eligible participants were allocated to either the TAU condition or intervention according to the clinical judgement of the therapist who provided the initial assessment. Further details on the rationale of the allocation procedure can be found in the trial protocol [29]. The progression from registration through the feasibility trial, with reasons for exclusions, is presented in Figure 1.

### Demographics

Demographic information (eg, age, gender, and course information) was provided by clients when they completed the online registration form to be considered for counseling. Following online registration, clients attended the counseling service for the initial assessment to determine whether counseling would be an appropriate treatment option. Clients who were eligible for counseling and met the trial eligibility criteria attended a research interview and provided written consent for their demographic information to be added to the data collected during the trial (eg, clinical data).

### Measures

The time frame for administering measures has been detailed in the trial protocol [29] in the form of a Standard Protocol Items: Recommendations for Interventional Trials (SPIRIT) diagram.

#### Generalized Anxiety Disorder–7 and Patient Health Questionnaire–9

Anxiety and depression were measured with the 7-item Generalized Anxiety Disorder (GAD-7) [30] and the 9-item Patient Health Questionnaire (PHQ-9) [31]. These measures were in addition to the clinical measures used by the counseling service to allow comparisons with external counseling services. The GAD-7 and PHQ-9 were administered at baseline, 3 months, and 6 months following recruitment. Items refer to the previous 2 weeks and are scored on a 4-point Likert scale (0 = not at all, 3 = nearly every day) whereby higher scores indicate higher severity. The PHQ-9 is a reliable measure of depression severity that has been validated against other measures of depression [31-33]. The GAD-7, which is widely used alongside the PHQ-9, is a reliable screening tool for generalized anxiety with good construct, criterion, factorial, and procedural validity.

#### Clinical Outcomes in Routine Evaluation Outcome Measure–10

The 10-item Clinical Outcomes in Routine Evaluation Outcome Measure (CORE-10) [34] was administered at the initial clinical assessment (preintervention) and at every counseling session to measure changes in general psychological functioning. Items refer to the previous week and are scored on a 5-point Likert scale (0 = not at all, 4 = most or all of the time), whereby higher scores indicate higher symptom severity. The CORE-10 is a shortened version of the 34-item Clinical Outcomes in Routine Evaluation–Outcome Measure (CORE-OM) [35], which has been used extensively in mental health services in the United Kingdom. The 10-item version has been validated against the CORE-OM, is sensitive to change, and can be used to determine whether a client meets membership of a clinical population (score of ≥10).

#### Counseling Center Assessment of Psychological Symptoms–62

The Counseling Center Assessment of Psychological Symptoms (CCAPS-62) [36] is a measure developed specifically for the student college population and was administered with the CORE-10 at the initial clinical assessment (preintervention) to measure changes in student-specific mental health concerns. In line with its intended use, the shortened version CCAPS-34 was used alongside the CORE-10 at every counseling session after the initial assessment. The two versions are used interchangeably, and items refer to the previous 2-week period. Items are scored on a 5-point Likert scale (0 = not at all like me, 4 = extremely like me), whereby higher scores indicate higher symptom severity. The CCAPS-62 monitors changes in the following areas: depression, generalized anxiety, social anxiety, academic distress, eating concerns, hostility, substance use, family distress, and suicide ideation. The CCAPS-34 also monitors these areas except for family distress, which does not appear, and substance use, which is replaced with alcohol use. The CCAPS-62 has been validated against the CORE-10 using a UK student sample, which replicated the psychometric factor structure and internal reliability of the CCAPS [37].

### Clinical Interventions

All participants received face-to-face counseling in line with standard practice, which included a wait period of 3 to 5 working days for the initial clinical assessment and 8 to 10 days between ongoing therapy sessions. Sessions were 50 minutes in length, and the frequency of sessions was determined through client-therapist discussions as well as the usual disruptions from the academic timetable (eg, student time-off during Easter, summer, and course placements). Two clinical interventions were available through the trial: counseling alone (TAU) and counseling supplemented with guided use of a well-being app (intervention).

### Counseling Supplemented With Well-Being App (Intervention)

In addition to the standard level of care, counseling sessions in the intervention were supplemented with feedback on client use of a well-being app. Clients were encouraged to use the app independently between counseling sessions with the intention to review app exercises with their therapist during each face-to-face counseling session. Therapists were provided with tablet computers to allow clients to access their app account and review their progress. Through this process, therapists reviewed clients’ app activity and together they decided which activities could be beneficial for clients to use between sessions.

App features included (1) daily behavior monitoring (eg, for mood, sleep, exercise, alcohol consumption, medication use, and time spent outside); (2) reflective thinking exercises with guided CBT, mindfulness, and positive visualization; (3) guided relaxation with breathing and meditation; (4) peer-led support through anonymous online communities and private groups; and (5) tracking short-term and long-term goals. Between counseling sessions, the app provided daily prompts to encourage participants to log their mood and behavior. However, any additional activity required clients to go into the app and select an exercise—for example, the exercise that was suggested during their last counseling session. Therapists did not have access to clients’ app activity except during face-to-face counseling sessions if clients decided to show therapists their activity. Therapists were encouraged to prompt the decision to review app activity; however, the decision was ultimately that of the clients.

### Selection of Well-Being App

There are many smartphone apps that offer tools for improving well-being through a range of common features typically based on CBT and mindfulness. The quality of mental health apps was assessed in a recent systematic review, and the top-ranking apps, starting with the highest quality, were (1) HealthyMinds, (2) AnxietyCoach, (3) Moodkit, (4) Pacifica, and (5) Self-help for Anxiety Management (SAM) [38]. This quality assessment incorporated 16 recommendations, and these top 5 apps have the following properties: based on CBT; address anxiety and low mood; permit the reporting of thoughts, feelings, and behaviors; offer reminders; offer inbuilt activities; and contain a visual log to monitor progress. To aid the decision of selecting a well-being app for the purpose of this trial, the following criteria were applied: (1) applicable to university students, (2) demonstrates potential to be integrated with face-to-face counseling, (3) available on iOS and Android platforms, and (4) offers a range of features overlapping with other well-being apps.

Based on these criteria, the Pacifica app [39] was selected and evaluated by a volunteer sample of students and in-house therapists before it was implemented in the feasibility trial. It is important to note that while this study used a specific app, the app and its functions are representative of well-designed apps, and the feasibility trial is not intended to be an evaluation of Pacifica per se. Annual app subscriptions were purchased and provided to participants as unique gift codes. All payments were subject to the standard fee for public users and no financial incentives or waivers were provided by the Pacifica development team.

### Therapists Delivering the Interventions

All therapists were accredited by the British Association for Counselling and Psychotherapy (BACP) or the UK Council for Psychotherapy and were employed by the university counseling service. Six therapists (4 intervention, 2 TAU) were assigned to support the trial and delivered either the intervention or TAU condition, based on their preference. Therapists received additional training specific to the intervention they were allocated; this training has been described elsewhere [29]. Therapists were provided with a competence framework [40] for the university and college counseling context together with the most recent service clinical handbook to ensure best practice. Clinical practice was reinforced throughout the trial with regular team meetings and optional daily drop-in sessions for members of the counseling team to query issues with the onsite researcher.

### Primary Outcomes (Feasibility)

The yield of the feasibility trial was a series of primary and secondary outcomes relating to a range of components that aimed to inform a definitive trial. The primary outcomes were recruitment duration, treatment preference, and randomization acceptability. The recruitment period for a definitive trial was estimated from this study by exploring the required time needed to reach 40 participants while also considering therapist availability. Treatment preference was determined by asking participants to indicate their preferred treatment out of the two options available: counseling alone versus counseling with guided use of a well-being app. Clients were further asked whether their assigned treatment condition affected their decision to join the trial—for example, if they were not assigned to their preferred condition. Similarly, clients were asked whether being randomized would increase their likelihood of withdrawing from the trial. For brevity, additional criteria listed in the protocol have not been reported [29].

### Secondary Outcomes (Clinical Effects)

The secondary outcomes were clinical outcomes, clinical change across counseling sessions, clinical change at 3 months and 6 months following recruitment, and reliable and clinically significant improvement (RCSI) at 3 months and 6 months. Clinical outcomes were calculated as the difference between each counseling session and the difference between baseline, 3-month, and 6-month measures for the following mental health indicators: anxiety (GAD-7), depression (PHQ-9), psychological functioning (CORE-10), student-specific symptoms such as academic distress, social anxiety, substance use, and eating concerns (CCAPS-34 and 62). RCSI was calculated in line with standard methods [41]. According to these methods, clients must have had contact with the counseling service twice and their precounseling scores on the PHQ-9/GAD-7 must have been in the clinical range. To meet the threshold for RCSI, individual scores on both measures needed to meet each of two criteria: extent of change significantly greater than measurement error (reliable change) and postintervention score below the clinical cutoff, indicating nonclinical status (clinical change). For brevity, additional secondary criteria in the protocol have not been reported.

### Statistical Analysis

Analyses were predominantly descriptive (eg, mean, standard deviation, minimum, and maximum) to characterize the study population and outline various feasibility measures. As the sample size was not powered to detect significant differences between TAU and intervention groups, data were primarily used to summarize group outcomes to reveal preliminary trends and inform the design of a pilot trial. Independent *t* tests were conducted where appropriate to clarify whether group differences were significant (eg, clinical severity at intake, across conditions).

## Results

### Recruitment

The recruitment period was from February to June 2016; however, recruitment was not active for the whole period because the service experienced several disruptions. These included disruptions from the academic calendar (eg, Easter break), breaks in staff contracts, and therapists with full caseloads (ie, no counseling slots available). Recruitment aimed to simulate the natural demands from the service to reduce service disruption. Participants were recruited into the trial when therapists had available slots rather than protecting counseling slots for trial participants. Similarly, the time between sessions was dependent on therapist availability as it would in routine practice. Recruitment ended with 38 participants (intervention, n=20; TAU, n=18); the trial entered the academic summer period when students typically leave university before reaching the recruitment goal of 40 participants. Attention then focused on collecting follow-up measures with existing participants.

### Treatment Preference and Randomization Acceptability

Of the TAU participants, 11 preferred their allocated condition because it required less work than the intervention, 6 participants preferred the intervention because it offered additional support, and 1 had no preference. Of participants in the intervention group, 1 participant preferred the TAU condition for requiring less input, 10 preferred the intervention, and 9 had no preference. Despite their treatment preferences, 37 participants reported they would still have joined the trial if they had been randomized to the alternative condition; 1 participant in the intervention group claimed they would likely have withdrawn if allocated to the TAU group as they were specifically interested in using the app alongside counseling.

### Intervention Fidelity

Intervention fidelity was assessed using anonymized transcripts of counseling audio recordings from clients in the intervention group. A total of 45 recordings (12 clients across 5 therapists) were available and transcribed for analysis. Transcripts were scored to assess the following criteria: (1) number of times app was discussed, (2) duration of app discussion, (3) whether therapist reviewed client app use, (4) number of app features therapist suggested, and (5) missed opportunities to discuss client app use. All transcripts were rated by author EB, and 24% (11/45) of transcripts were also rated by a blinded independent researcher. Interrater reliability analysis revealed substantial to almost perfect agreement across raters with kappa values in the range of .81 to 1.00 [42]. On average, therapists reviewed client app use, held brief discussions to review client app activity (lasting 2 to 5 minutes per counseling session), and provided advice based on client feedback. Therapists rarely missed an opportunity to review client app use; however, 2 of the 5 therapists were more likely to miss an opportunity and one was less likely to initiate app discussion (ie, relied on client to raise app discussion). There were no clear associations between therapist checklist scores and therapeutic modality (eg, CBT psychotherapist versus humanistic).

### Baseline Demographic Data

Forty-two participants were initially recruited by therapists and attended the research consent session to determine eligibility (Figure 1). Two participants, both female, were excluded because one scored <10 on the PHQ-9 and GAD-7 measures and another had been referred for online self-help instead of counseling. A further 2 participants left the university after the initial service assessment and were excluded from the trial. Of the remaining 38 participants, 20 were allocated to the intervention group (10/20, 50%, were women) and 18 were allocated to the TAU group (12/18, 67%, were women; allocation procedure can be found elsewhere [29]). The average age in the TAU group was 23 years (SD 4.11, minimum 19, maximum 32); the intervention group was younger with a mean age of 21 years (SD 3.24, minimum 19, maximum 35). Of the total sample, 79% (30/38) were undergraduate, 8 were postgraduate (taught = 3, research = 5), 30 were students studying in their birth country or from the European Union and 8 students were from an international university and visiting a UK university as part of their course. Participants studied in the following faculties: arts and humanities (6/38, 16%); engineering (9/38, 24%); medicine, health, or dentistry (3/38, 8%); science (10/38, 26%); and social science (10/38, 26%).

### Baseline Clinical Scores

Table 1 shows that both the TAU and intervention groups met the eligibility criteria of moderate clinical threshold (≥10) on the PHQ-9 and GAD-7 at baseline. The TAU group scored higher than the intervention group for both PHQ-9 and GAD-7, but independent samples *t* tests revealed that these differences were not significant (PHQ-9: *t*_36_=1.53, *P*=.14; GAD-7: *t*_36_=0.82, *P*=.42). The group means for CORE-10 and CCAPS distress index met moderately severe clinical threshold, and the TAU group scored higher than the intervention group for the CCAPS distress index; however, this difference was not significant (*t*_34_=1.11, *P*=.55). The remaining group differences between the CCAPS-62 subscales were also not significant (depression: *t*_34_=1.42, *P*=.11; generalized anxiety: *t*_34_=1.58, *P*=.12; social anxiety: *t*_34_=1.84, *P*=.08; academic distress: *t*_34_=1.11, *P*=.17; eating concerns: *t*_34_=1.22, *P*=.23; family distress: *t*_34_=1.19, *P*=.55; hostility: *t*_34_=1.11, *P*=.85; substance use: *t*_34_=1.38, *P*=.13).

**Table 1:** Baseline scores on clinical measures on the Physical Health Questionnaire–9, Generalized Anxiety Disorder–7, Clinical Outcomes in Routine Evaluation Outcome Measure–10, and Counseling Center Assessment of Psychological Symptoms–Distress Index across intervention and treatment as usual groups from a feasibility trial.

Measures		Baseline clinical scores across groups						*P* value^a^
		Treatment as usual			Intervention			
		N	Mean (SD)	Min-Max	N	Mean (SD)	Min-Max	
PHQ-9^b,c^		18	17.58 (5.99)	10-26	20	15.00 (4.71)	10-24	.14
GAD-7^c,d^		18	13.75 (4.56)	5-19	20	13.92 (4.27)	7-20	.42
CORE-10^e,f^		11	22.64 (6.44)	13-32	17	23.18 (5.41)	15-34	.55
**CCAPS-62^g,h^**								
	Depression	16	2.70 (0.69)	1.31-3.54	20	2.30 (0.92)	0.08-3.54	.11
	Generalized anxiety	16	2.70 (0.85)	1.44-4.00	20	2.26 (0.80)	0.11-3.56	.12
	Social anxiety	16	3.00 (0.59)	1.86-3.86	20	2.53 (0.90)	0.00-3.86	.08
	Academic distress	16	2.79 (0.98)	1.40-4.00	20	2.34 (0.93)	0.60-3.40	.17
	Eating concerns	16	1.49 (0.96)	0.00-3.33	20	1.13 (0.82)	0.00-2.78	.23
	Family distress	16	1.11 (0.79)	0.17-2.83	20	1.05 (0.87)	0.00-2.83	.55
	Hostility	16	1.57 (0.93)	0.29-2.86	20	1.59 (0.95)	0.14-3.57	.85
	Substance use	16	0.77 (1.20)	0.00-4.00	20	0.93 (0.94)	0.00-3.50	.13
	Distress index	16	2.68 (0.66)	1.42-3.75	20	2.40 (0.82)	0.25-3.80	.55

^a^*P* value from independent samples *t* test comparing treatment as usual and intervention group means.

^b^PHQ-9: Physical Health Questionnaire, 9-item.

^c^PHQ-9 and GAD-7 clinical boundaries: 0-5 = mild, 6-10 = moderate, 11-15 = moderately severe, 16-20/21 = severe.

^d^GAD-7: Generalized Anxiety Disorder, 7-item.

^e^CORE-10: Clinical Outcomes in Routine Evaluation Outcome Measure, 10-item.

^f^CORE-10 clinical boundaries: 0-5 = healthy, 5-10 = low, 10-15 = mild, 15-20 = moderate, 20-25 = moderately severe

^g^CCAPS-62: Counseling Center Assessment of Psychological Symptoms, 62-item.

^h^CCAPS-Distress Index clinical boundaries: 12.1 = low, 2.15 = elevated.

### Clinical Change

**Figure 2:**
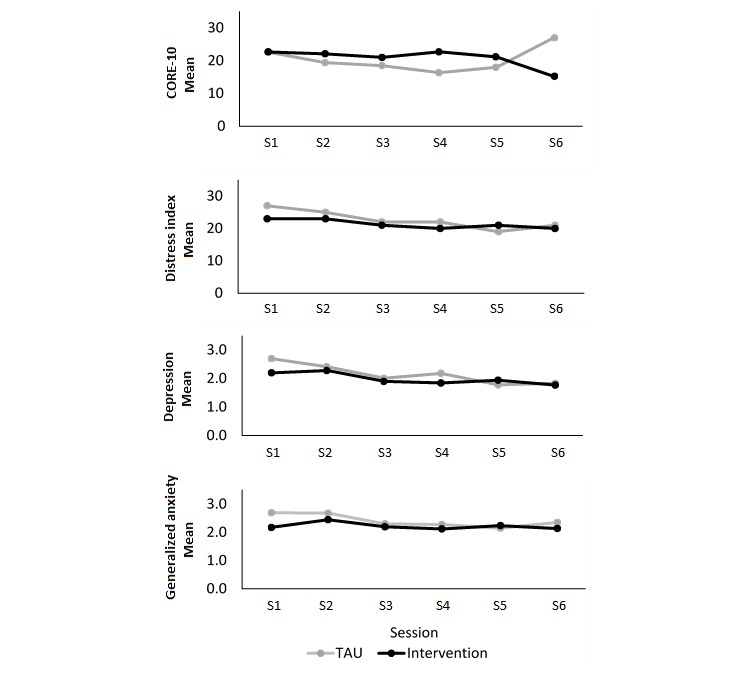
Clinical change scores for CORE-10 and CCAPS subscales across therapy sessions 1 to 6 for the TAU and intervention groups of the feasibility trial. CORE-10: Clinical Outcomes in Routine Evaluation Outcome Measure, 10-item; CCAPS-62: Counseling Center Assessment of Psychological Symptoms, 62-item; TAU: treatment as usual.

**Figure 3:**
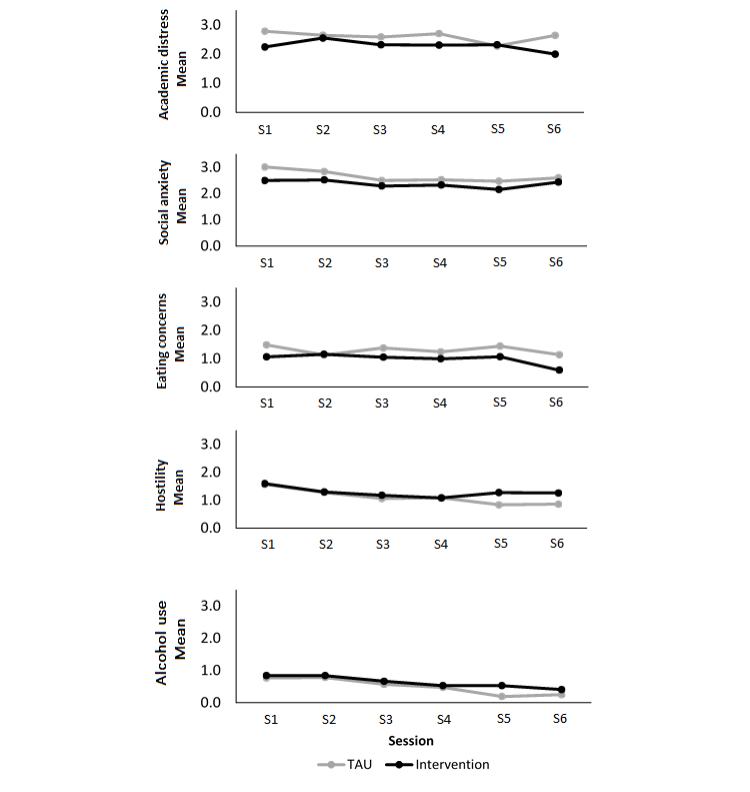
Clinical change scores for remaining CCAPS subscales across therapy sessions 1 to 6 for the TAU and intervention groups of the feasibility trial. CCAPS-62: Counseling Center Assessment of Psychological Symptoms, 62-item; TAU: treatment as usual.

Participants in the intervention group on average received 5 counseling sessions (mean 4.73 [SD 1.62], minimum 2, maximum 9), and TAU participants received 4 (mean 5.29 [SD 1.73], minimum 2, maximum 10). The average waiting period between counseling sessions was approximately 4 days longer for the TAU group than the intervention group (TAU mean 19.50 [SD 16.43], minimum 4, maximum 75; intervention mean 15.92 [SD 6.91], minimum 7, maximum 31). As shown in Figures 2 and 3 both the TAU and intervention groups decreased their distress scores across counseling sessions for all CCAPS subscales but not for the CORE-10. By session 6, both groups had decreased their scores on social anxiety, alcohol use, and hostility to a large enough extent to leave the clinical group they met at session 1 (ie, below the elevated or low clinical boundary). This was also true for the intervention group mean for academic distress and eating concerns.

### Pre-Post Clinical Change

**Figure 4:**
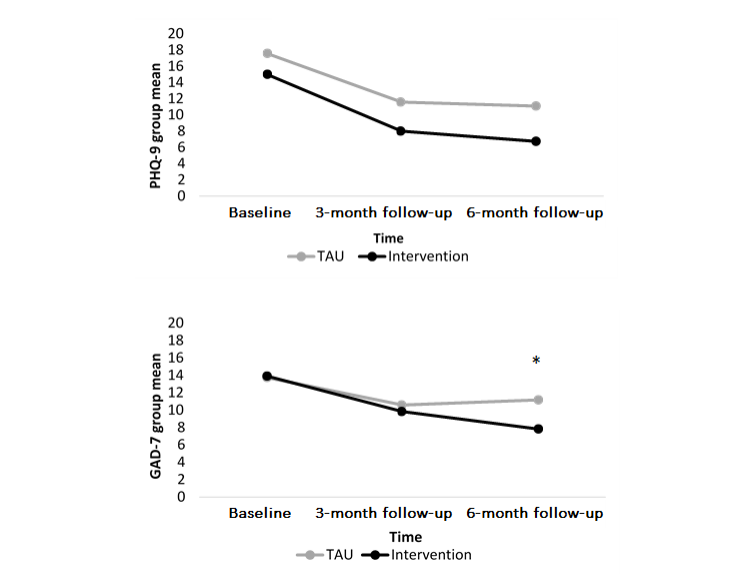
PHQ-9 and GAD-7 scores across participants from the intervention and TAU groups at 3-month and 6-month follow-up measures. PHQ-9: Patient Health Questionnaire, 9-item; GAD-7: Generalized Anxiety Disorder, 7-item; TAU: treatment as usual.

Clinical change was calculated as the difference between clinical scores for measures administered at baseline, 3 months, and 6 months following recruitment. All participants had completed counseling by the 6-month follow-up period. However, clients in both groups were still receiving treatment during the 3-month follow-up period. Depression and anxiety were measured at baseline, 3 months, and 6 months with the PHQ-9 and GAD-7. Figure 4 shows that PHQ-9 scores of both groups decreased between the baseline and 3-month follow-up period and continued to decrease slightly at the 6-month follow-up. GAD-7 scores of both groups also decreased between the baseline and 3-month follow-up but to a lesser extent than the PHQ-9 scores. By the 6-month follow-up, GAD-7 scores of the TAU clients had increased whereas GAD-7 scores of the intervention clients continued to decrease, and this difference was significant (GAD-7: *t*_22_=3.46, *P*=.002). The difference between PHQ-9 scores at follow-up, however, was not significant (PHQ-9: *t*_22_=1.30, *P*=.21).

### Reliable and Clinically Significant Improvement

All clients in the feasibility trial met the criteria to calculate RCSI in line with the established criteria [41], which required clients to obtain a PHQ-9 ≥10 at baseline for depression, have decreased this score by ≥6 points, and have a post-counseling score of <10 (below the clinical threshold). The criteria for RCSI for anxiety required clients to obtain a GAD-7 ≥8 at baseline, have decreased this score by ≥5 points and have a post-counseling score of <8. If client post-counseling scores decreased by ≥6 points on the PHQ-9 or ≥5 points on the GAD-7 but their scores are not below the clinical thresholds, they have made a reliable improvement. Tables 2 and 3 show that at 6 months, 75% (9/12) of TAU clients met RCSI on the PHQ-9 and 17% (2/12) met RCSI on the GAD-7. Fewer intervention clients met RCSI on the PHQ-9 by 6 months compared with TAU clients. By contrast, more intervention clients met RCSI on the GAD-7 by 6 months, and 50% (6/12) met RCSI at 3 months.

**Table:** Individual treatment as usual participant scores on the Physical Health Questionnaire–9 and Generalized Anxiety Disorder–7 at baseline and 3- and 6-month follow-up measures with reliable and clinically significant improvement indicators.

Measure across time		PHQ-9^a^			GAD-7^b^		
		Baseline	3 month FU^c^	6 month FU	Baseline	3 month FU	6 month FU
**Individual**							
	11	22	18	16^d^	16	13	14
	12	10	4^e^	3^e^	5	3	4
	15	16	10^d^	10^d^	11	9	10
	17	13	6^e^	5^e^	10	1^e^	3^e^
	19	10	10	8	12	11	11
	26	23	20	20	14	15	16
	28	23	20	21	18	17	17
	31	25	10^d^	14^d^	19	13^d^	15
	34	26	14^d^	13^d^	19	15	15
	35	18	12^d^	12^d^	17	15	15
	36	12	8	5^e^	16	11^d^	10^d^
	41	13	7^e^	6^e^	8	4	4
**Aggregate**							
	Mean	17.58	11.58	11.08	13.75	10.58	11.17
	SD	5.99	5.38	5.96	4.56	5.28	5.06
	Minimum	10	4	3	5	1	3
	Maximum	26	20	21	19	17	17
	RI^f^ count, n (%)	—^g^	7 (58)	9 (75)	—	3 (25)	2 (17)
	RCSI^h^ count, n (%)	—	3 (25)	4 (33)	—	1 (8)	1 (8)

^a^PHQ-9: Physical Health Questionnaire, 9-item.

^b^GAD-7: Generalized Anxiety Disorder, 7-item.

^c^FU: follow-up.

^d^RI scores decreased by ≥6 points on the PHQ-9 and ≥5 points on the GAD-7.

^e^RCSI scores meet RI criteria and have postcounseling scores below the clinical cut-points.

^f^RI: reliable improvement.

^g^Not applicable, as RI and RCSI scores must be calculated from two data points.

^h^RCSI: reliable and clinically significant improvement.

**Table 3:** Individual intervention participant scores on the Physical Health Questionnaire–9 and Generalized Anxiety Disorder–7 at baseline and 3- and 6-month follow-up measures with reliable and clinically significant improvement indicators.

Measure across time		PHQ-9^a^			GAD-7^b^		
		Baseline	3 month FU^c^	6 month FU	Baseline	3 month FU	6 month FU
**Individual**							
	02	23	6^d^	7^d^	17	11^e^	8^e^
	03	16	9^d^	5^d^	7	3	3
	04	15	6^d^	6^d^	20	18	15^e^
	05	12	10	8	18	11^e^	9^e^
	06	18	9^d^	8^d^	15	10^e^	10^e^
	08	10	6	7	8	5	5
	10	11	9	9	12	15	5^d^
	13	12	7	5^d^	13	8^e^	7^d^
	14	24	10^e^	8^d^	19	12^e^	10^d^
	22	16	8^d^	5^d^	12	7^d^	6^d^
	23	13	8	7^d^	10	6	6
	27	10	8	6	16	12	10^e^
**Aggregate**							
	Mean	15.00	8.00	6.75	13.92	9.83	7.83
	SD	4.71	1.48	1.36	4.27	4.28	3.21
	Minimum	10	6	5	7	3	3
	Maximum	24	10	9	20	18	15
	RI^f^ count, n (%)	—^g^	6 (50)	8 (67)	—	6 (50)	9 (75)
	RCSI^h^ count, n (%)	—	5 (42)	8 (67)	—	1 (8)	4 (33)

^a^PHQ-9: Physical Health Questionnaire, 9-item.

^b^GAD-7: Generalized Anxiety Disorder, 7-item.

^c^FU: month follow-up.

^d^RCSI scores meet RI criteria and have post-counseling scores below the clinical cut-points.

^e^RI scores decreased by ≥6 points on PHQ-9 and ≥5 points on GAD-7.

^f^RI: reliable improvement.

^g^Not applicable, as RI and RCSI scores must be calculated from two data points.

^h^RCSI: reliable and clinically significant improvement.

## Discussion

### Principal Findings

This study reports on the feasibility, benefits, and challenges of supplementing counseling with a well-being app for students experiencing anxiety or depression. The trial assessed essential design elements to inform how well the trial was implemented into a service and whether the intervention was acceptable. The aim of the trial was to explore the feasibility and acceptability of supplementing counseling with a well-being app and whether this, in turn, had positive clinical outcomes.

### Primary Feasibility Outcomes

The trial was conducted in a naturalistic setting and embedded into a student counseling service with early engagement from the therapeutic team. The design of the trial and subsequent intervention was developed with high stakeholder engagement from across the higher education counseling sector including service users, providers, and experts in the field of trial design. This collaborative approach aimed to minimize service disruption, optimize acceptability, and streamline the recruitment capacity of the service. Recruitment mirrored routine practice as far as possible in that therapist availability to take on new clients was a key factor. Recruitment delays were anticipated because of the reliance on therapist availability, but further delays were introduced from breaks in staff contracts and therapists managing full caseloads.

A challenge of embedding a trial into practice is implementing the additional research requirements without negatively impacting the service. Randomization was a concern for this reason, and identifying randomization acceptability can help to alleviate disruption. Only one participant reported that they would have withdrawn if they had been randomized to the alternative condition, suggesting that randomization would be acceptable in a fully powered trial. While client preferences did not impact participation, evidence suggests that discussing and measuring client preference is associated with higher treatment satisfaction, engagement, and clinical outcomes [43]. Clients perceiving desirable components in both conditions also highlights a benefit of using active control groups compared with traditional wait-list control groups. This design choice is particularly relevant for the student counseling context as research suggests that comparing an active treatment against a wait-list control group is not an accurate comparison when testing short-term psychotherapy for depression or social anxiety [44].

### Secondary Feasibility Outcomes

Clients in both groups decreased their levels of distress and clinical severity on all outcome measures as they progressed through counseling. This gradual reduction across counseling was observed despite clients in both groups entering counseling with moderately severe scores. The symptom profile of clients was multifaceted and particularly elevated for social anxiety, academic distress, depression, and generalized anxiety. These symptoms complement findings on the CCAPS from other UK-derived student counseling samples [37]. The elevated symptoms of social anxiety, depression, and generalized anxiety mimic three prevalent conditions reported in primary care with the additional impact on academic performance. In our counseling sample, the reduction of clinical scores across counseling provides preliminary evidence on the effectiveness of student counseling embedded in higher education.

In addition to the clinical improvement observed during counseling, depression scores for the majority of clients across both groups reliably improved by the end of the trial (ie, at 6-month follow-up). The addition of the well-being app had a larger impact on anxiety than depression as clients using the app reliably decreased their anxiety scores sooner and to a greater extent than clients who only received counseling. The group difference in anxiety is noteworthy because it was achieved despite clients entering the trial with similar levels of anxiety across the groups. This suggests that the addition of the app contributed to the group difference observed at the end of the trial, suggesting that counseling augmented with the app may be more effective than counseling alone at reducing anxiety. Given the aforementioned pressures on student counseling services, this finding has important implications for counseling services and the wider student population. The use of app-augmented counseling may be a way for counseling services embedded in higher education institutions to do more with less.

### Limitations and Future Research

Feasibility trials are, by nature, underpowered and explorative because they provide the groundwork for clinical trials and inform key design elements. Research embedded into practice must also be pragmatic to ensure that trials are sufficiently implemented with minimal disruption to the service. These necessary design decisions introduced variability across the trial and potentially diluted the quality of the intervention. The delivery of the intervention needed to be flexible for several reasons. For instance, in-house counseling is variable across therapists and institutions because services are designed to respond to the unique needs of their students. Counseling is also more variable than other forms of therapy (eg, CBT), and at the time the trial was being designed, there was limited direction from clinical manuals. Another necessity for the flexibility of the trial was to ensure that client risk was at the forefront of any design decision as the intervention had not been previously tested. That is, supplementing short-term counseling with guided use of a well-being app in the student context was a new concept, and there was little research or clinical experience that could anticipate risk. It was, therefore, a collective decision not to randomize at this stage, and this decision limits the implications of the subsequent feasibility trial.

Blended face-to-face and app approaches have proven effective in other domains [45], and student counseling seems an appropriate setting in which to develop this approach further. For example, evidence from RCTs has shown internet and app-based interventions to be particularly effective at reducing depressive symptoms [46]. These findings have been demonstrated across a range of technologies and therapeutic approaches including guided internet CBT with text message reminders, internet-delivered mindfulness, and an internet-delivered prevention program comprising self-monitoring and relaxation exercises [47]. Additional lines of research could include the introduction of an app precounseling, which could help to bridge a treatment gap by offering simple guided exercises through the convenience and privacy of a mobile phone. Offering well-being apps while waiting for counseling has the potential to prepare students by providing a means to self-monitor moods, thoughts, and behaviors to take to counseling. Future research would benefit from adopting clear descriptions of blended interventions involving counseling in order to build the evidence base and inform training and guidelines as much of the literature is dominated by blended interventions offering CBT. Regarding the design and implementation of the feasibility trial, future research should consider the need for pragmatic research methodologies to explore outcomes from natural settings that translate to routine practice.

### Conclusion

Mobile apps for health and well-being have become ubiquitous and have been developed quicker than research has been able to evaluate their quality [17]. This disconnect has led to a large proportion of apps becoming available without the necessary evidence to validate the quality or efficacy of app content. However, increased awareness of this evidence gap has sparked the development of new quality frameworks, assessment criteria, and a shared acceptance to co-create new technologies with clinical expertise and embedded evaluations [48]. The future of digital technology is promising for addressing some of the treatment barriers of traditional therapeutic interventions and provides an innovative solution to extend existing services. Offering therapeutic technologies as a low-level preventative measure for subclinical symptoms of depression is particularly promising for the student population and has been shown to be effective. Using apps as an adjunct to counseling also offers a unique solution to addressing some of the attrition issues observed in student counseling services by encouraging self-guided support between face-to-face sessions. This blended approach in our study was shown to be acceptable and feasible and showed potential to maintain clinical improvement on anxiety following the completion of brief counseling.

## Abbreviations

BACP: British Association for Counselling and Psychotherapy
CASELOAD: Counseling plus Apps for Students Experiencing Levels Of Anxiety or Depression
CCAPS-62: Counseling Center Assessment of Psychological Symptoms, 62-items
CORE-10: Clinical Outcomes in Routine Evaluation Outcome Measure, 10-item
CORE-OM: Clinical Outcomes in Routine Evaluation–Outcome Measure
DSM-IV: Diagnostic and Statistical Manual of Mental Disorders, 4th Edition
GAD-7: Generalized Anxiety Disorder, 7-item
iCBT: internet-based cognitive behavioral therapy
NHS: National Health Service
PHQ-9: Physical Health Questionnaire, 9-item
RCSI: reliable and clinically significant improvement
RCT: randomized controlled trial
SAM: Self-help for Anxiety Management
SPIRIT: Standard Protocol Items: Recommendations for Interventional Trials
TAU: treatment as usual
